# Machine Learning Based Object Classification and Identification Scheme Using an Embedded Millimeter-Wave Radar Sensor

**DOI:** 10.3390/s21134291

**Published:** 2021-06-23

**Authors:** Homa Arab, Iman Ghaffari, Lydia Chioukh, Serioja Tatu, Steven Dufour

**Affiliations:** 1 École Polytechnique de Montréal, Montréal, QC H3T 1J4, Canada; i_ghafari@ymail.com (I.G.); lydia.chioukh.1@ens.etsmtl.ca (L.C.); steven.dufour@polymtl.ca (S.D.); 2 Institut National de la Recherche Scientifique (INRS), Montréal, QC H2X 1E3, Canada; Serioja.Ovidiu.Tatu@inrs.ca

**Keywords:** doppler frequency, in-phase/quadrature demodulator, machine learning, millimeter-wave, multi-class SVMs, metronome, radar cross section (RCS), wavelet scalogram

## Abstract

A target’s movements and radar cross sections are the key parameters to consider when designing a radar sensor for a given application. This paper shows the feasibility and effectiveness of using 24 GHz radar built-in low-noise microwave amplifiers for detecting an object. For this purpose a supervised machine learning model (SVM) is trained using the recorded data to classify the targets based on their cross sections into four categories. The trained classifiers were used to classify the objects with varying distances from the receiver. The SVM classification is also compared with three methods based on binary classification: a one-against-all classification, a one-against-one classification, and a directed acyclic graph SVM. The level of accuracy is approximately 96.6%, and an F1-score of 96.5% is achieved using the one-against-one SVM method with an RFB kernel. The proposed contactless radar in combination with an SVM algorithm can be used to detect and categorize a target in real time without a signal processing toolbox.

## 1. Introduction

The analyses of the received electromagnetic signal from different targets have been of great importance in many research and engineering topics. Accurate computational methods in classification and identification of targets are very much desired. Autonomous self-driving car [1], intelligent robots [2], smart home devices [3], and diagnosing disease [4] are just some of the domains that usually target detection and target classification play an important role. Wireless radar sensing using millimeter wave signals is proven as an effective tool for various purposes [5,6,7]. For instance, in [8], a millimeter wave radar sensor is presented for early-stage detection of melanoma skin cancer. The proposed sensor has ability to detect melanomas in its early and most treatable stages with the accuracy on the order of tens of microns. In [9,10], a 77-GHz six-port sensor is designed for accurate near-field displacement and Doppler measurements with high accuracy, at a reasonable cost. In [11], a millimeter wave radar sensor is studied for medical signal detection. In [12], they developed fully coherent, solid state, FMCW radar systems operating at 94 and 340 GHz, which is suitable for micro-Doppler and vibrometry studies with the sensitivity of 1 micron in range measurements. In [13], they proposed to use of Ultra Wideband (UWB) radars and audio processing techniques for eavesdropping applications in various environments.

The millimeter-wave radar sensor can measure phase, frequency and relative amplitude variations of the reflected signal from the target, with respect to a reference signal derived from the transmitted continuous wave (CW) signal. Various parameters, such as object dimensions, shapes, material properties, positions, and speeds of movement could be extracted from the received signal. However, the effect of many static and dynamic objects in the environment would increase the uncertainty in the classification results. In such cases, machine learning algorithms can be utilized for extracting information about the target from the huge number of recorded data sets [14,15,16]. In machine learning, the classification problem is solved using the supervised and unsupervised learning algorithms. One of the popular supervised learning algorithms is the SVM algorithm [17,18,19,20,21,22].

Support Vector Machines (SVMs) are very powerful tools in pattern recognition and data mining applications and it has many usages in various applications. Breathing disorder recognition [4], human-vehicle classification [23], human gait classification [24], spectral signature classifications [25], hand-gesture recognition [26], and image change detection [27] are some studies that demonstrated using supervised learning algorithms. The SVM algorithm was mainly formulated for problems of two binary classifications, and the extension to multi-class problems is required for our research. Using a set of the binary classifiers one against all (OAA), one against one (OAO), and directed acyclic graph (DAG) are the approach are introduced in the following [28,29,30]. SVM method is a strong tool when the dataset is relatively small, here for four shapes. For multiple target classification with big amount of datasets, multi-layer neural networks and deep learning techniques can give higher classification accuracy [31,32,33,34]. They require a large number of training data and cost lots of time to train the deep neural networks that can generalize well.

For the radar prototype, two 16 dBi gain 32-element patch antenna arrays have been used in the transmitter and receiver modules. Metronome experiments are placed in one meter from the sensor and the displacement of the metronome’s pendulum is detected in various *x* and *y*-axis directions for various speeds. The time domain Doppler frequency in comparison with the metronome pendulum position relative to the baseline; and spectrogram of Doppler frequencies are presented and discussed. The information gathered and results produced can be beneficial for remote monitoring such as vital sign, human fall detection, human activity recognition, intelligent target recognition and many other industrial and biomedical applications.

In most previous studies [35,36,37,38,39,40], the shape of a micro-Doppler signature is used to classify targets. In this work, we propose to use the spectrum of baseband radar signals to classify targets. From the spectrum of Doppler signals, many features can be extacted including the target’s physical properties, cross section, range, and motion. To the best of our knowledge, we are the first to perform such evaluation for target classification based on the spectrum of I, and Q signals using a 24 GHz radar sensor. The 24 GHz radar sensor can be used to detect targets for short, and middle-range applications and has high accuracy for detecting range and frequncy (the mean error for the frequency is ±0.01 Hz and for the range is ±10 micron).

This paper is organized as follows: Section 2 presents the analytical analyses for the 24 GHz CW radar for a metronome’s pendulum swinging on the baseline of the sensor, followed by simulation illustrations of the swinging pendulum. The experimental investigations are presented in Section 3 which includes the Doppler results for swinging pendulum in x and y directions. To the best of our knowledge, this is the first work that applied SVM techniques as a classifier for real-time millimeter wave radar signals. In Section 4, a supervised machine learning tools (SVM) are trained by the recorded data to classify the shape of targets into four different categories. The trained classifiers were used to predict shape of objects by comparing the magnitude of power in baseband signals for various target ranges. The conclusion and future works are given in the last section.

## 2. Mathematical and Simulation Analyses

A metronome experiment is performed using the 24 GHz commercial CW Doppler radar front-end [41] which is shown in Table 1.

The signal transmitted by the waveform generator is given by

(1) $$\begin{array}{c} V_{tx}\left(t\right)=A_{t}\cos\left(2\pi ft+\theta (t)\right), \end{array}$$

where *f* is the frequency of the transmitted signal, θ(t) is the phase noise from the RF generator, and $A_{t}$ is the amplitude of the transmitted signal. The received signal can then be approximated by

(2) $$V_{rx}\left(t\right)=A_{r}\cos\left(2\pi ft-\frac{4\pi R(t)}{\lambda }+\theta (t-\frac{2R(t)}{c})\right),$$

where $A_{r}$ is the amplitude of the receiver signal, R(t) is the distance of the target, λ is the wavelength of the carrier, and *c* is the speed of light. The transmitted signal is used as a local oscillator (LO) signal to down-convert the received signal. The baseband in phase (*I*) and quadrature-phase (*Q*) downconversion results are the following signals

(3) $$\begin{array}{c} I\left(t\right)=A_{I}\cos\left(\frac{4\pi R(t)}{\lambda }+\theta \left(t\right)-\theta (t-\frac{2R(t)}{c})\right),\\ Q\left(t\right)=A_{Q}\sin\left(\frac{4\pi R(t)}{\lambda }+\theta \left(t\right)-\theta (t-\frac{2R(t)}{c})\right). \end{array}$$

where $A_{I}$, and $A_{Q}$ are the amplitude of the baseband *I*, and *Q* signals, respectively. The residual phase noise, θ(t)−θ(t−2R(t)/c), is negligible due to the coherent nature of the sensor. In fact, both the TX and the LO signals have the same source and phase noise, which can cancel each other [42]. Equation (3) shows that the movement of target (R(t)) modulated the phase of received signal as

(4) $$\phi \left(t\right)=\frac{4\;\pi \;R(t)}{\lambda }.$$

Doppler frequncy can be obtained by the time drivative of phase changes of Equation (3)

(5) $$f_{D}\left(t\right)=\frac{2f_{0}}{c}\;\frac{\partial }{\partial t}R\left(t\right).$$

**Figure 1:**
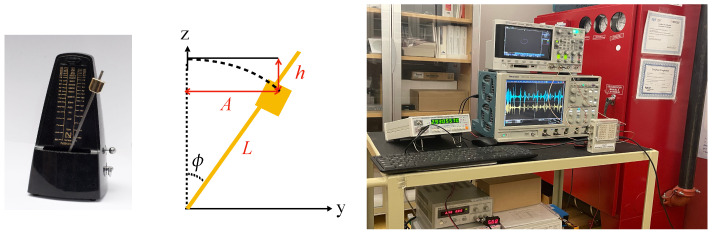
Metronome’s pendulum geometry, and experimental setup.

In this experiment, the metronome is used as a target. The metronome’s pendulum geometry is shown in Figure 1.

The maximum linear movement of metronome’s pendulum, *A*, and the maximum height of the metronome’ pendulum, *h*, are given by

(6) $$A=L\;\sin\left(\phi_{0}\right),\;h=L\left(1-\;\cos\left(\phi_{0}\right)\right).$$

The angular displacement changes by time when metronome pendulum starts to swim. These time variation can be defined by, $\phi \left(t\right)=\phi_{0}\sin\left(\omega_{0}t\right)$, where $\omega_{0}$ is the angular frequency given by

(7) $$\omega_{0}=\sqrt{\frac{m_{1}l_{1}-m_{2}l_{2}}{m_{1}l_{1}^{2}+m_{2}l_{2}^{2}}g},$$

where a metronome is treated as a double-weighted pendulum with the parameters defined as: $m_{1}$ is the mass of the metronome’s movable weight, $l_{1}$ is the distance of $m_{1}$’s center of mass from the rotation point, $m_{2}$ is the mass of the fixed counterweight, and $l_{2}$ the distance of $m_{1}$’s center of mass from the rotation point, and g is gravitational force of restitution. More details about metronome angular frequency can be found in [43]. If the sensor is placed in the *x*-axis with the range of *R*, then the pendulum movement in *x*, *y* and *z* directions are

(8) $$\begin{array}{c} R_{x}\left(t\right)=R,\\ R_{y}\left(t\right)=L\;\sin\left(\phi_{0}\sin\left(\omega_{0}\;t\right)\right),\\ R_{z}\left(t\right)=L\;(1-\cos\left(\phi_{0}\sin\left(\omega_{0}\;t\right)\right). \end{array}$$

If the sensor is placed in the *y*-axis with the range of *R*, then the pendulum movement in *x*, *y* and *z* directions are

(9) $$\begin{array}{c} R_{x}\left(t\right)=0,\\ R_{y}\left(t\right)=R+2\;|L\;\sin\left(\phi_{0}\sin\left(\omega_{0}\;t\right))\right|,\\ R_{z}\left(t\right)=L\;(1-\cos\left(\phi_{0}\sin\left(\omega_{0}\;t\right)\right), \end{array}$$

then, the instantaneous range from the sensor to the metronome’s pendulum is given by

(10) $$R\left(t\right)=\sqrt{R_{x}{\left(t\right)}^{2}+R_{y}{\left(t\right)}^{2}+R_{z}{\left(t\right)}^{2}}.$$

As shown in Equations (8) and (9), the instantaneous range of targets are combination of constant and sinusoidal parts. For the sinusoidal part of R(t), a special technique is required to recover accurate information about baseband signal phase shift. Therefore, to simplify the analysis, and without any loss of generality, we consider the sinusoidal movement to be as $R\left(t\right)=\phi_{0}\sin\left(\omega_{0}\;t\right)$. The received *I*, and *Q* signals can be defined by their Taylor expansion of the Bessel function order *n*, $J_{n}\left(x\right)$ [44,45]. Therefore, the baseband *I*, and *Q* signals can be reconstructed as

(11) $$\begin{array}{c} I\left(t\right)=A_{I}J_{0}\left(\frac{4\pi \phi_{0}}{\lambda }\right)+2A_{I}\sum_{n=1}^{\infty }J_{2n}\left(\frac{4\pi \phi_{0}}{\lambda }\right)\cos\left(2n\omega_{0}t\right)\\ Q\left(t\right)=2A_{Q}\sum_{n=0}^{\infty }J_{2n+1}\left(\frac{4\pi \phi_{0}}{\lambda }\right)\sin\left(\left(2n+1\right)\omega_{0}t\right) \end{array}$$

The harmonics in experimental baseband signals validate this mathematical expansion which is the result of non-linear property of the cosine transfer function. These harmonics can also be used to extract ehe important information desired about the target velocity and ranges. It also has to be considered that the metronome moving parts have different cross-sections which create modulations in the amplitude of the baseband signals.

## 3. Experimental Verifications

The 24 GHz front-end radar, presented in Table 1, was used to perform metronome experiments. The experimental setup is shown in Figure 1. This radar included a low noise microwave amplifier (LNA) which allowed us to increase the level of the input signal. It was an embedded small size radar, internally generating a 24 GHz signal. This radar could be integrated with various types of devices for a multitude of applications. As a target, a NIKKO standard mechanical metronome with various oscilation speed (40 to 208 oscillations/minute or 0.666–3.466 Hz) was considered. Tektronix DPO7054 Oscilloscope was used to display time domain I and Q baseband signals, and Agilent DSO-X 2024A oscilloscope shows the I versus Q signals (Lissajous curve) at the same time. These double displays allowed us to visualize not only time domain signals (to measure amplitudes and/or period of signals) but also an intuitive phase/frequency variation, as in the case of a Doppler effect (a point in the I/Q plane moving on a circle or a spiral shape in clockwise or counterclockwise direction on the screen).

The tune voltage was set between 0 to 5 V to adjust the transmitted signal frequency on 24 GHz band (1 GHz from 23.5 to 24.5 GHz), useful also in FM modulation if needed. The transmitted microwave frequency could be measured using TFC-3000 digital universal frequency counter (displayed frequency multiplied by 8192). This was an internal feature of the radar which allowed measurement of the transmitted frequency using a low-cost equipment. The measurements were carried out in an anechoic chamber to remove the effect of other objects.

**Figure 2:**
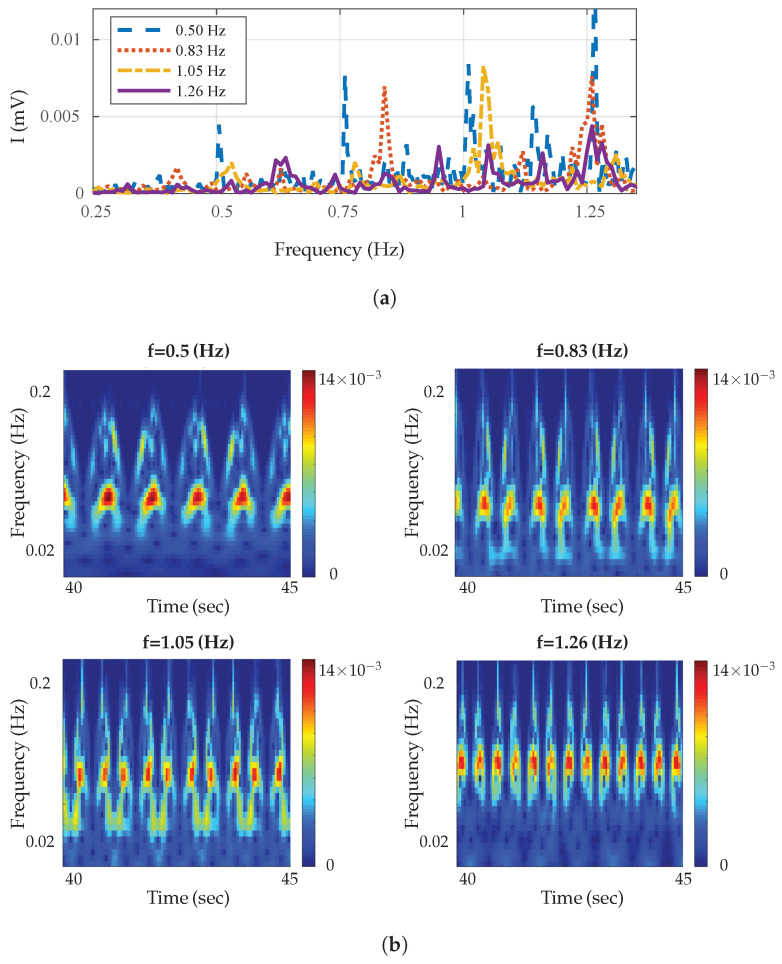
Baseband signals reflected from the metronome. (**a**) Spectrum of baseband signals at various frequencies. (**b**) Wavelet magnitude scalograms of baseband signals for metronome at various frequencies.

**Figure 3:**
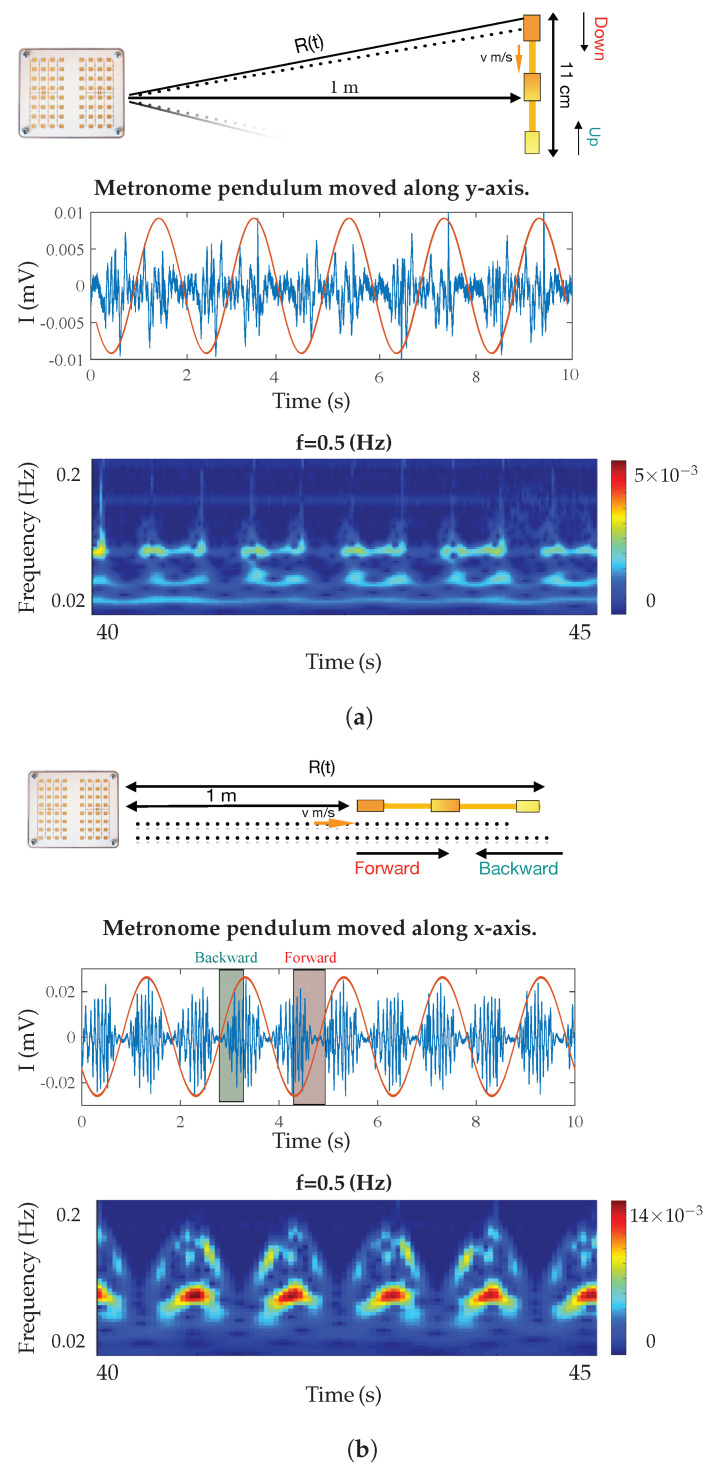
Baseband signals reflected from metronome while its pendulum is oscilated along *x* and *y* axis. (**a**) Wavelet scalograms of baseband signals for metronome pendulum moved along *y*-axis. (**b**) Wavelet scalograms of baseband signals for metronome pendulum moved along *x*-axis.

In order to validate the test bench set-up, in a typical radar measurement, a first experiment was done. A mechanical oscillating target, in this case a calibrated metronome, was placed at the range of 1 m from the sensor and baseband signals were analyzed. Its pendulum oscillated in two different directions along the *x* and *y* axis. The measurement results for various frequencies are shown in Figure 2a,b with a ±0.01 Hz accuracy. Figure 2b shows the time–frequency characteristics for the Doppler frequencies generated using a continuous wavelet transforms (CWT) spectrogram on Matlab. The minimum and maximum scales were determined automatically based on the energy spread of the wavelet in frequency and time. As shown in Figure 2b, the Doppler value started from near zero and increased with the increase of the metronome pendulum tangential velocity until the pendulum reached the maximum ranges, where the Doppler value dropped to zero at the maximum range. The same scenario happened in a backward motion where the Doppler value was zero at starting point and maximum range and increased again as range decreased; therefore, the Doppler value was zero again at the start point where we had the minimum displacement. The baseband *I* signal and wavelet scalogram of metronome when its pendulum swung along the *y* axis is plotted in Figure 3a, and along the *x* axis is plotted in Figure 3b. The Doppler frequency for both cases was 0.5 Hz which is shown with red color. In each case we had different scalogram and baseband signal due to differences in range variations. The measurement results were in good agreement with the expected values as indicated on the metronome and analytical results. The results show that we could differentiate swinging direction based on the phase change and amplitude of the received signal. The proposed radar could be used in many applications like heart rate detection, human motion behavior monitoring, material classification, etc. In the next section, a machine learning algorithm is used to classify shape of targets based on their radar cross sections (RCSs).

## 4. Supervised Machine Learning

In this section, SVM techniques are used to classify various target shapes (1: square of area 324 cm${}^{2}$, 2: triangle of area 626.4 cm${}^{2}$, 3: circle of area=268.8 cm${}^{2}$, and 4: rectangular of area=656.7 cm${}^{2}$ metal plates). For this purpose, the power of baseband signals are measured and analyzed for targets at various ranges (0.5 to 1 m). The magnitude received power at various ranges was influenced by the presence of the materials and it could be used for classifying them. For making SVM classification, a small interval range of frequency from 2.4 to 2.5 Hz was chosen which provided 1000 data sample for each class. Different type of machine learning algorithms could be utilized for extracting information about the target from the huge number of recorded data sets. Here, the SVM algorithm was used which is one of the more powerful machine learning algorithms. Support vector machines (SVMs) are based on the supervised learning methods associated with a nonhypprobabilistic binary linear classifier that are usually employed for classification (SVChyp–Support Vector Classification) or regression (SVR—Support Vector Regression) problems. More importantly, one of the benefits is its ability to adjust the capacity of learning machine according to the scale of a specific problem by optimal margin classifier method [46,47,48]. In this method, SVM separates data across the decision boundary, named hyper plane f(x)=0, using solving a constrained quadratic optimization problem. It is worth mentioning that the decision boundary can be a line or hyper plane depending on the input feature. In other words, if two features are needed, then the boundary decision would be a line. On the other hand, if more than two features are needed, then the boundary condition may be a hyperplane. In SVC, the prime problem is to separate two distinguished classes. To this end, the given input data $x_{i}$, (i=1,2,...N) is composed of objects with +1 and −1 labels. Positive classes are located on one side and negative classes are located on another side. Then, the distances between two classes should be maximized perpendicular to the decision boundary, i.e., the maximum margin. Linear decision function will be the following form

(12) $$\begin{array}{c} f\left(x\right)=W^{T}x+b=\sum_{i=1}^{N}W_{i}x_{i}+b \end{array}$$

where *N*, *W*, and *b* are the number of samples, N-dimensional weighting vector, and scalar, respectively. Besides, linear classification formulation can be extended to nonlinear SVM using the Kernel function. To be more exact, let consider $x_{i}\in R^{m}$ and $y_{i}\in \left[\pm 1\right]$, with nonlinear function ϕ, input vector $x_{i}$ is mapped to ϕ(x). By solving quadratic optimization problem, the optimal classifier is obtained as follows:

(13) $$\begin{array}{c} W\left(\lambda \right)=\sum_{i=1}^{l}\lambda_{i}-\frac{1}{2}\sum_{j=1}^{l}\left[\lambda_{i}\lambda_{j}y_{i}y_{j}\phi \left(x_{i}\right)\phi \left(x_{j}\right)\right] \end{array}$$

**Figure 4:**
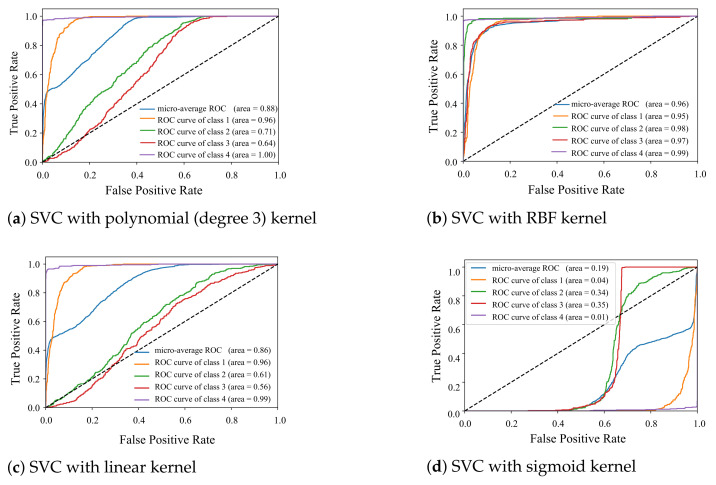
SVM of the measured data into four shap categories using different kernel functions.

Besides, $K\left(x_{i},x_{j}\right)=\left(\phi \left(x_{i}\right),\phi \left(x_{j}\right)\right)$ is considered to be the kernel if it can satisfy the Mercer’s condition. The most common kernels that have been used are linear, polynomial, radial basis function, and sigmoid [49]. It is worth to mention that there is no analytical study to choose the optimal kernel. In the current research, we applied different kernel functions in SVC method and the receiver operating characteristic (ROC) curves are presented in Figure 4. For evaluating classifier output quality, receiver operating characteristic (ROC) curves are plotted for various kernel functions. ROC curves typically feature a true positive rate on the *y*-axis, and a false positive rate on the *x*-axis. This means that the top left corner of the plot is the ideal point and the larger area under the curve (AUC) is usually showing better classification accuracy. The results show that RBF gave us more accuracy in comparison to the other kernel functions. The RBF kernel function was

(14) $$\begin{array}{c} K\left(x_{i},x_{j}\right)=\exp-\frac{∥x_{i}-x_{j}{∥}^{2}}{2\alpha^{2}} \end{array}$$

where α controls the width of the RBF kernel. For SVC classifier using an RBF kernel, two parameters γ and *C* had to be defined. γ is a parameter of the RBF kernel and can be thought of as the ‘spread’ of the kernel and therefore the decision region. C is a parameter of the SVC learner and is the penalty for misclassifying a data point. For this specific problem, c=1 and γ=1 gave us acceptable results which are shown in Figure 4b. The approximation based on the uniform and monotonic basis function such as sigmoid cannot be optimal since sigmoid has non-zero values and the rate of converging of these functions is slow. On the other hand, using non-uniform functions better approximation can be obtained. RBFs can train faster since the output weight of each hidden node can converge quickly by overcoming the output of other nodes by providing input values for that node [50].

Since SVMs were originally designed for binary problems, several methods were proposed to extend binary SVMs to solve multi-classification problems. One approach for doing so is to reduce the single multi-class problem into multiple binary problems. Multi-class pattern recognition problems are commonly solved using a combination of binary SVMs and a decision strategy to decide the class of the input pattern. Each SVM is independently trained. In this section, we applied One-Against-All (OAA), One-Against-One (OAO), and Directed Acyclic Graph (DAG) multi-class SVM methods which are based on binary classifiers [19,48].

**Figure 5:**
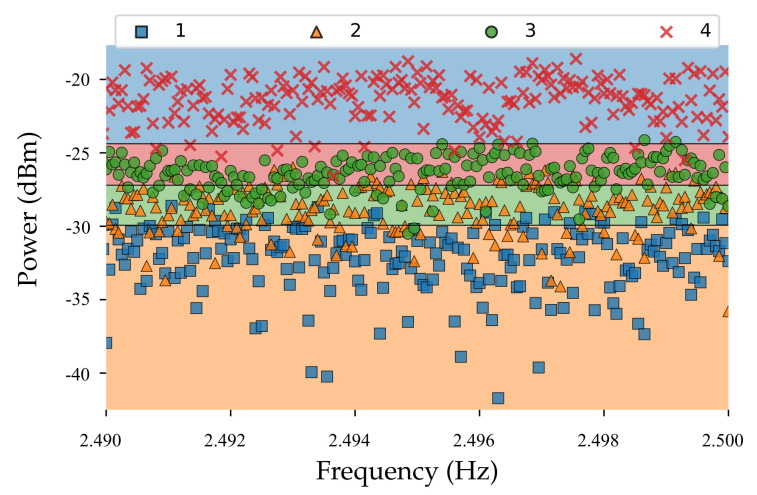
Shape classification using RBF kernel SVC methods (square (1), triangle (2), circle (3), and rectangular (4)).

The results illustrated in the Table 2 show the accuracy and F1-score (weighted average of the precision and recall) of the multi-class SVM methods were close and OAO obtained the best results in comparison to OAA, and DGA methods. Likewise, training time and testing time were three times higher in case of OAA methods. The class separation using RBF kernel SVC method is illustrated in Figure 5. The classification was performed for four specific geometries; however it was extendable for detecting more targets. Morever for more different target, more advanced machine learning and deep learning algorithms could be applied to classifying big data set into categories.

## 5. Conclusions

This paper presented a 24 GHz radar sensor for extracting information about various parameters of a target, displacement, and Doppler frequency. For evaluating the accuracy of the radar sensor, a metronome is used as a target and the reflected signals are analyzed for detection metronome’s pendulum parameters. Moreover, supervised machine learning tools (SVMs) are trained by the recorded data to classify the shape of targets into four different categories. SVM classification performance is also compared for three methods based on binary classifications: one-against-all, one-against-one, and directed acyclic graph SVM (DAGSVM). The accuracy level of 96.6% and F1-score of 96.5% is achieved using one-against-one SVM Methods with RFB kernel function. One limitation of our current classification techniques is that when we have multiple targets using SVC algorithm wont achieve a high classification accuracy. For future work, we will study convolutional neural network (CNN) deep learning method for building dynamic models that are appropriate to identify multiple objects.

## Figures and Tables

**Table 1:** 24 GHz radar sensor parameters and prototype [41].

Characteristic	Value	Radar Prototype
Frequency Range	23.5–24.5 GHz	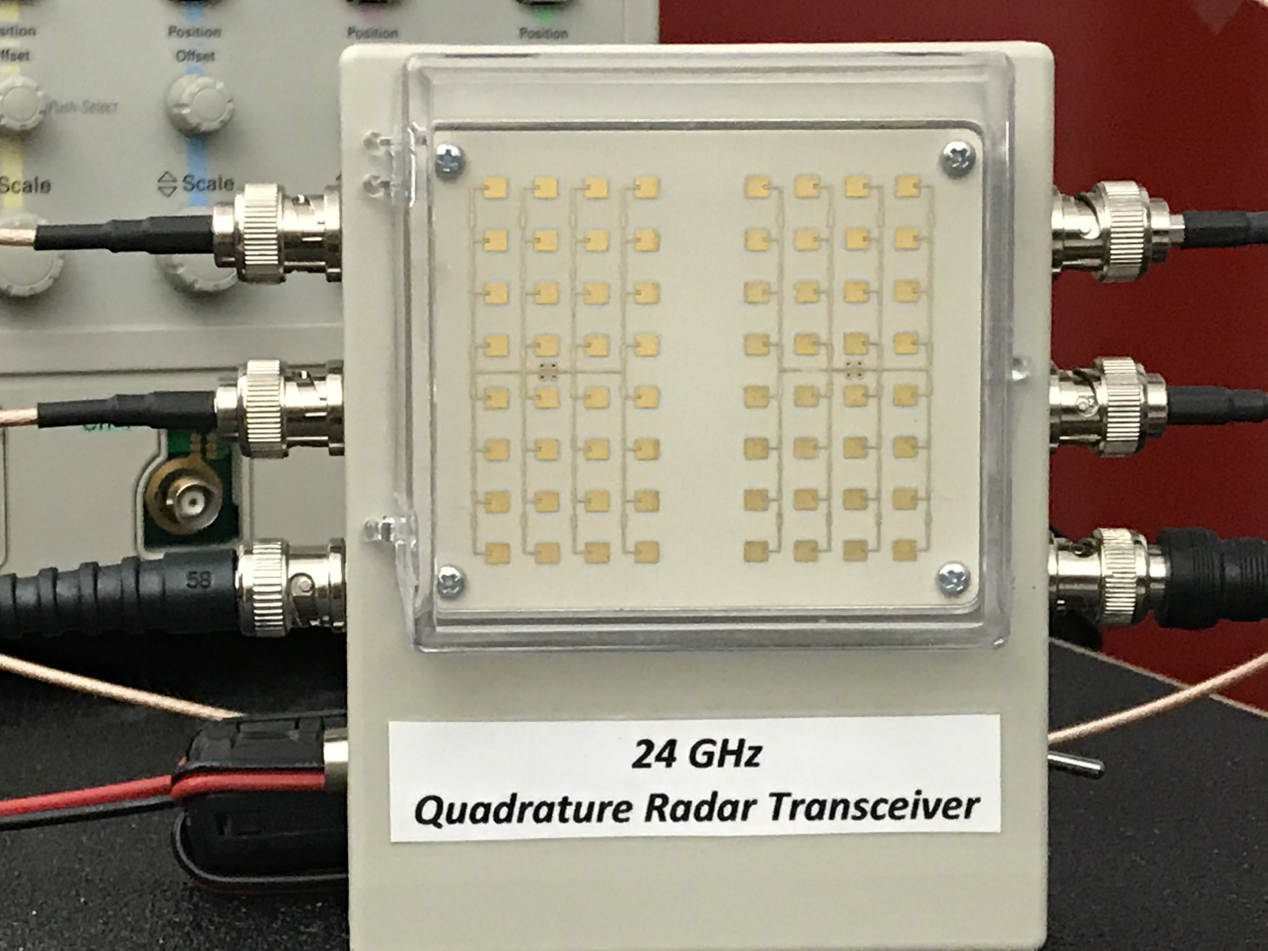
Horizontal −3 dB beamwidth	$24^{\circ }$	
Vertical −3 dB beamwidth	$16^{\circ }$	
Side Lobe Level (H-plane)	−29 dB	
Side Lobe Level (E-plane)	−11 dB	
Tx Gain	17 dBi	
Rx Gain	16 dB	Antenna radiation patterns
Ambient Temperature Range	−25 to +85 ${}^{\circ }$C	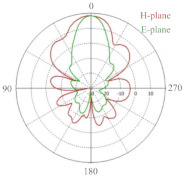
Storage Temperature Range	−40 to +105 ${}^{\circ }$C	
IF 1st Cascade	+20 dB	
IF 2nd Cascade	+14 dB	
Deviation	1000 MHz (0 to +5 V)	

**Table 2:** Comparing various SVMs multiclass methods.

SVM Methods: RFB kernel, (C = 1, and γ=1)			
	OAA	OAO	DAG
Accuracy	96.32	96.62	96.61
F1-score	96.28	96.55	96.59

## Data Availability

Data sharing not applicable.
